# Synovial T cell hyporesponsiveness to myeloid dendritic cells is reversed by preventing PD-1/PD-L1 interactions

**DOI:** 10.1186/s13075-014-0497-x

**Published:** 2014-11-30

**Authors:** Frederique M Moret, Kim MG van der Wurff-Jacobs, Johannes WJ Bijlsma, Floris PJG Lafeber, Joel AG van Roon

**Affiliations:** Department of Rheumatology & Clinical Immunology, University Medical Center Utrecht, PO Box 85500, 3508 GA Utrecht, The Netherlands; Laboratory of Translational Immunology, University Medical Center Utrecht, PO Box 85090, 3508 AB Utrecht, The Netherlands

## Abstract

**Introduction:**

The aim of this study was to investigate PD-1/PD-L1 involvement in the hyporesponsiveness of rheumatoid arthritis (RA) synovial fluid (SF) CD4 T cells upon stimulation by thymic stromal lymphopoietin (TSLP)–primed CD1c myeloid dendritic cells (mDCs).

**Methods:**

Expression of PD-1 on naïve (Tn), central memory (Tcm) and effector memory (Tem) CD4 T cell subsets was assessed by flow cytometry. PD-L1 expression and its regulation upon TSLP stimulation of mDCs from peripheral blood (PB) and SF of RA patients were investigated by quantitative RT-PCR and flow cytometry. The involvement of PD-1/PD-L1 interactions in SF T cell hyporesponsiveness upon (TSLP-primed) mDC activation was determined by cell culture in the presence of PD-1 blocking antibodies, with or without interleukin 7 (IL-7) as a recognized suppressor of PD-1 expression.

**Results:**

PD-1 expression was increased on CD4 T cells derived from SF compared with PB of RA patients. TSLP increased PD-L1 mRNA expression in both PB and SF mDCs. PD-L1 protein expression was increased on SF mDCs compared with PB mDCs and was associated with T cell hyporesponsiveness. Blockade of PD-1, as well as IL-7 stimulation, during cocultures of memory T cells and (TSLP-primed) mDCs from RA patients significantly recovered T cell proliferation.

**Conclusion:**

SF T cell hyporesponsiveness upon (TSLP-primed) mDC stimulation in RA joints is partially dependent on PD-1/PD-L1 interactions, as PD-1 and PD-L1 are both highly expressed on SF T cells and mDCs, respectively, and inhibiting PD-1 availability restores T cell proliferation. The potential of IL-7 to robustly reverse this hyporesponsiveness suggests that such proinflammatory cytokines in RA joints strongly contribute to memory T cell activation.

## Introduction

Rheumatoid arthritis (RA) is characterised by progressive joint inflammation that results in tissue damage [1]. This is strongly dependent on CD4 T cell production of Th1 (interferon γ) and Th17 cytokines (interleukin 17 (IL-17)) [2-5]. Activation and differentiation of CD4 T cells to become Th1 or Th17 cells is strongly regulated by antigen-presenting cells such as dendritic cells (DCs) [6]. Several types of DCs are known to circulate in human blood. They are characterised by high expression of human leucocyte antigen (HLA) class II molecules and the absence of lineage markers (CD3, CD19, CD14, CD20, CD56 and glycophorin A). Human blood DCs can be divided into at least three subtypes (plasmacytoid DCs and two types of myeloid or classical DCs (mDC1 and mDC2)) [7,8], based on the blood-derived DC antigen (BDCA) molecules [9,10]. BDCA-1 (CD1c) identifies the mDC1 subset, which comprises potent activators of CD4 T cells, whereas mDC2 cells, identified by expression of BDCA-3 (CD141), more potently activate CD8 T cells [7,9,10]. In this respect, it is important to note that the characterisation of mDC1 cells by CD1c is more specific than the previously used and more broadly expressed marker, CD11c [7,9]. CD1c mDCs are abundantly present in joints of RA patients, and these synovial fluid (SF)–derived mDCs have recently been demonstrated to have an extremely strong capacity to activate autologous peripheral blood (PB)–derived CD4 T cells [11].

Thymic stromal lymphopoietin (TSLP) has recently been considered as a potential trigger to activate CD1c mDCs in the joints of RA patients. TSLP cytokine levels are significantly increased in the SF of RA patients compared with SF of osteoarthritis patients [12,13]. TSLP has been demonstrated to potently activate TSLPR-expressing CD1c mDCs from SF to secrete enhanced levels of T cell–attracting chemokines and to strongly activate PB-derived CD4 T cells to induce Th1, Th17 and Th2 activity [13]. In addition, recently, TSLP and its receptor were also shown to enhance Th1- and Th17-mediated experimental arthritis and tissue destruction [14].

Because of the prominent role of CD4 T cells in arthritic processes and the potential of SF-derived mDCs and TSLP-primed mDCs to activate autologous PB-derived CD4 T cells, in this study we investigated the potential of these mDCs to activate autologous SF-derived CD4 T cells. An evident hyporesponsiveness of SF-derived CD4 T cells upon mDC or TSLP-primed mDC activation was observed. Several observations led us to investigate the role of programmed death 1 (PD-1) and its ligand interactions in this hyporesponsiveness, because ligation of PD-1 by PD-L1 or PD-L2 leads to inhibition of T cell proliferation [15,16]. First, our analysis of the gene expression profiles of TSLP-primed mDCs from RA patients revealed significant upregulation of PD-L1 and much higher expression levels compared with PD-L2. In addition, preliminary data had shown us that PD-L1 was upregulated on SF mDCs of RA patients. Third, data from previous studies [17] and ours indicated overexpression of PD-1 on synovial CD4 T cells of RA patients. Because IL-7 recently was shown to downregulate PD-1 expression on T cells [18] and because of the potent T cell stimulatory capacity of IL-7 [19], we also examined the role of IL-7 in the regulation of PD-1/PD-L interactions in hyporesponsiveness of synovial T cells. Our data demonstrate that hyporesponsiveness of synovial CD4 T cells in response to SF-derived CD1c mDCs is dependent on PD-1/PD-L1 interactions and is robustly reversed by IL-7.

## Methods

### Patients

SF was obtained from 13 RA patients during effusion of the knee and 70 ml of heparinized PB was collected from 16 RA patients. RA was classified according to the American College of Rheumatology criteria [20]. Ethical approval for this study was granted by the medical ethics committee of the University Medical Center Utrecht for the collection of patient samples in compliance with the Helsinki Declaration. All patients gave their written informed consent to participate.

### Cell isolation

Mononuclear cells (MNCs) were isolated from PB and SF by density centrifugation using Ficoll-Paque Plus (GE Healthcare, Uppsala, Sweden). Prior to MNC isolation, PB or SF was diluted 1:1 with RPMI 1640 medium (Gibco/Life Technologies, Grand Island, NY, USA) containing penicillin (100 U/ml), streptomycin (100 μg/ml) and glutamine (2 mM) (all from PAA Laboratories, Pasching, Austria). PB- and SF-derived CD1c mDCs and CD4 T cells were isolated from the MNC fraction by magnetic-activated cell sorting (MACS) using CD1c (BDCA-1)-positive dendritic cell and CD4^+^ T cell isolation kits (Miltenyi Biotec, Bergisch Gladbach, Germany). PB memory T cells were isolated from the CD4 T cell fraction by their lack of CD45RA expression using the CD45RA^+^ isolation kit (Miltenyi Biotec). Isolations were performed according to the manufacturer’s instructions.

### Cell cultures

All cells were cultured in RPMI GlutaMAX medium (Gibco/Life Technologies) supplemented with penicillin, streptomycin and 10% (vol/vol) GemCell human serum AB (Gemini Bio-Products, West Sacramento, CA, USA). The mDCs were cultured at a cell concentration of 0.5 × 10^6^ cells/ml with or without recombinant TSLP at 20 ng/ml (R&D Systems, Minneapolis, MN, USA) for 20 hours in Sarstedt tubes (Sarstedt, Nümbrecht, Germany) at 37°C.

The functional capacities of PB- and SF-derived mDCs were assessed by measuring activation of autologous CD4 T cells derived from PB and SF. For this purpose, isolated T cells were seeded in round-bottomed 96-well plates at a concentration of 0.25 × 10^6^ cells/ml and kept at 37°C in full culture medium before coculturing with the (TSLP-primed) mDCs. Washed TSLP-activated mDCs and unstimulated mDCs were added to the autologous T cells (mDC:T cell ratio 1:5 or 1:10) in triplicate in the absence of additional stimuli and cocultured for 6 days. To test the effects of PD-1 blockade or IL-7 effects in cocultures of mDCs and memory T cells from PB and SF, anti-PD-1 mAB (1 μg/ml; BioLegend, San Diego, CA, USA) or IL-7 (10 ng/ml, PeproTech, Rocky Hill, NJ, USA) was added at the start of the cocultures. Proliferation was measured by [^3^H]thymidine incorporation (1 μCi/well added during the last 18 hours of a culture period; PerkinElmer, Waltham, MA, USA).

### Flow cytometry

PD-1 expression on CD4 T cells and PD-L1 expression on CD1c mDCs of RA patients were analysed by flow cytometry using a FACSCanto II flow cytometer (BD Biosciences, San Jose, CA, USA). *Ex vivo* or cultured mDCs were stained with CD1c phycoerythrin (CD1c-PE; BD Biosciences), CD19 peridinin chlorophyll (CD19-PerCP; BioLegend) and CD274 (PD-L1)-APC (BioLegend). mDCs were gated as CD1c-positive and CD19-negative. *Ex vivo* CD4 T cells were stained with CD45RO fluorescein isothiocyanate (CD45RO-FITC; Dako, Glostrup, Denmark), CD27-APC (Invitrogen), CD279 (PD-1)-PE and CD4-PerCP (BioLegend) using isotype antibodies or autofluorescence as controls. All samples were analysed using FlowJo software (TreeStar, Ashland, OR, USA). To compare mean fluorescence intensity (MFI) values, the autofluorescence intensity was subtracted from the MFI of the stains to reveal true expression values.

### Real-time quantitative RT-PCR

To examine TSLP-mediated PD-L1 gene regulation in isolated CD1c mDCs from PB and SF of RA patients, CD1c mDCs (0.5 × 10^6^ cells/ml) were stimulated with 20 ng/ml recombinant TSLP (R&D Systems) or left untreated for 20 hours in Sarstedt tubes at 37°C. mDCs were collected and lysed in Buffer RLT (QIAGEN, Carpinteria, CA, USA) prior to RNA isolation. Total RNA was isolated using the RNeasy Mini Kit (QIAGEN) according to the manufacturer’s instructions. RNA quantity was measured by using a NanoDrop spectrophotometer (NanoDrop Technologies, Wilmington, DE, USA), and RNA quality was assessed on a 2100 Bioanalyzer (Agilent Technologies, Santa Clara CA, USA). cDNA synthesis was performed with an automated system (Caliper Life Sciences NV/SA, Teralfene, Belgium), starting with 70 ng of total RNA from each sample, as previously described in detail [21]. Gene expression was quantified by quantitative RT-PCR (RT-qPCR) performed in duplicate from 3 ng of cDNA in the presence of SYBR Select Master Mix (Life Technologies, Carlsbad, CA, USA) and 1.5 μM of specific primer pairs (PD-L1 forward: 5′-GCT GAA CGC ATT TAC TGT CAC-3′, PD-L1 reverse: 5′-TGT TCT TAT CCT CCA TTT CCC A-3′; GAPDH forward: 5′-ATG GGG AAG GTG AAG GTC G-3′, GAPDH reverse: 5′-GGG GTC ATT GAT GGC AAC AAT A-3′). The analyses were performed using the QuantStudio 12K Flex system (Life Technologies) with the following thermal cycle conditions: 95°C for 20 seconds, followed by 40 cycles of 95°C for 1 second and 60°C for 20 seconds. Gene expression values were calculated according to the comparative threshold cycle method [22] using the stably expressed GAPDH as an endogenous control. The value of each control sample was set at 1 and was used to calculate the fold change in target mRNA.

### Statistical analysis

Differences between conditions were assessed by paired-samples evaluation using the nonparametric Wilcoxon signed-rank test unless indicated otherwise. Unpaired data analysis was performed using the nonparametric Mann–Whitney *U* test. Data analysis was performed using SPSS software version 20.0 (SPSS, Chicago, IL, USA). Data were considered statistically significant at *P* < 0.05.

## Results

### Synovial CD4 T cells from RA patients are hyporesponsive to stimulation by (TSLP-primed) CD1c mDCs

**Figure 1:**
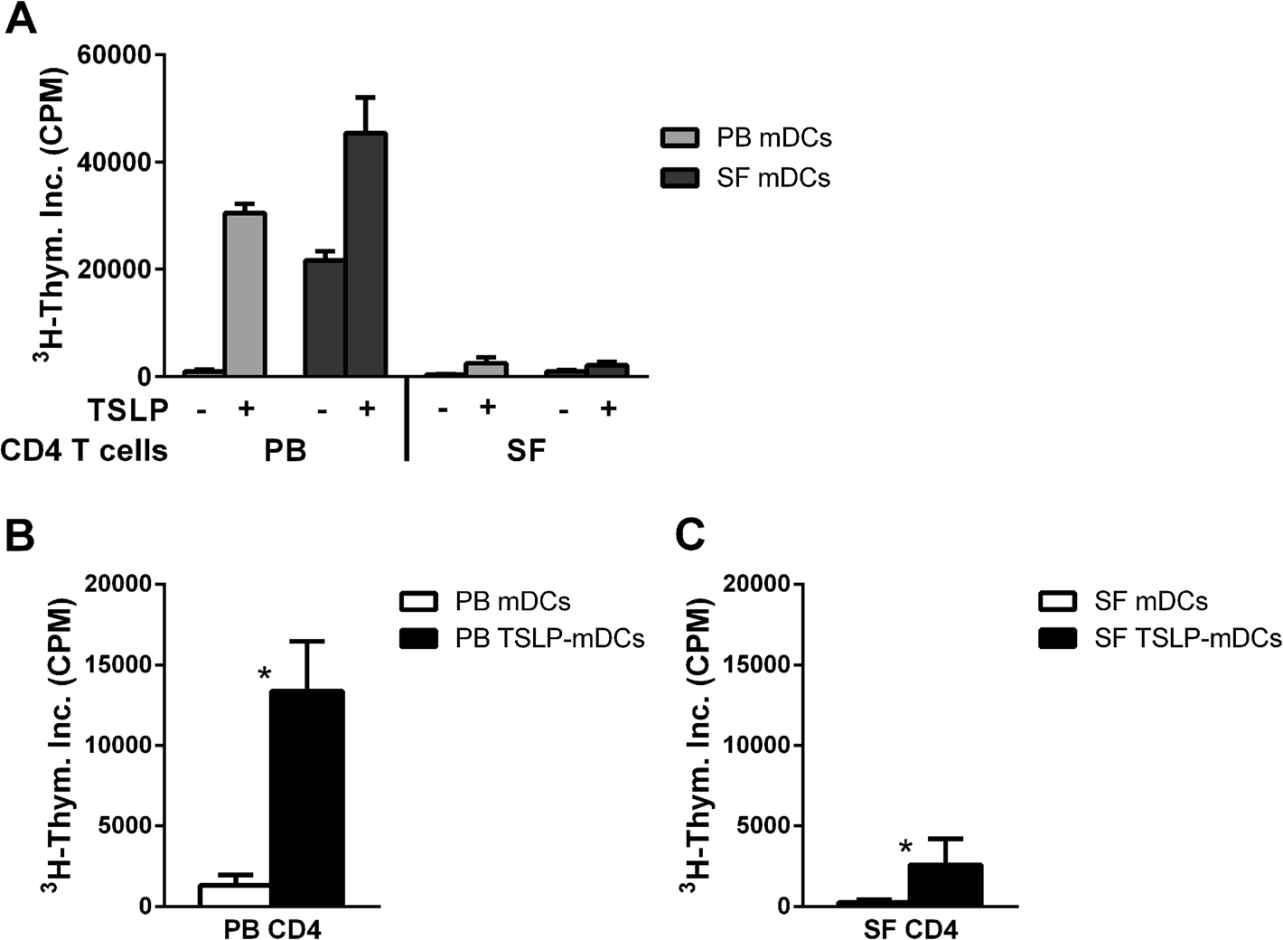
**CD4 T cells derived from synovial fluid of rheumatoid arthritis patients are hyporesponsive upon (TSLP-primed) myeloid dendritic cell stimulation in contrast to peripheral blood-derived CD4 T cells.** Thymic stromal lymphopoietin (TSLP)-primed myeloid dendritic cells (mDCs) from peripheral blood (PB) as well as both mDCs and TSLP-primed mDCs from synovial fluid (SF) of rheumatoid arthritis (RA) patients strongly activate PB-derived CD4 T cells, whereas SF-derived CD4 T cells are hyporesponsive upon (TSLP-primed) mDC activation. **(A)** mDC:T cell ratio 1:5, paired analysis of CD4 T cells and mDCs of a representative donor are shown. **(B)** mDC:T cell ratio 1:10 derived from PB of five RA patients. **(C)** mDC:T cell ratio 1:10 derived from SF of five RA patients. *P < 0.05 (statistically significant difference). CPM, counts per minute.

Myeloid DCs derived from SF of RA patients have a strong capacity to activate autologous PB-derived CD4 T cells (Figure 1A) [11]. In addition, TSLP-primed mDCs (TSLP-mDCs) from PB and SF strongly activate PB-derived T cells (Figure 1A) [13]. Whereas PB-derived CD4 T cells are strongly activated by TSLP-mDCs (Figures 1A and 1B), SF-derived CD4 T cells were hardly responsive to these *in vivo* and *in vitro* activated mDCs (Figures 1A and 1C).

Several recent observations by others and us indicated that programmed death-1 (PD-1)/PD ligand 1 (PD-L1) interactions could play a role in the regulation of synovial T cell hyporesponsiveness to (TSLP-primed) mDCs from RA patients. First, microarray analysis had shown that PD-L1 mRNA was highly expressed compared with PD-L2 mRNA expression and significantly upregulated upon TSLP activation in addition to upregulated costimulatory and antigen-presenting molecules such as CD80, CD86, HLA class II and CD1c (data not shown). In addition, preliminary data had shown us that PD-L1 was upregulated on SF mDCs of RA patients. Therefore, we set out to investigate the expression levels of PD-1 on T cells, PD-L1 on mDCs from PB and SF of RA patients and the capacity that this interaction has to regulate T cell responsiveness.

### Memory CD4 T cells from synovial fluid express robustly increased PD-1 levels

**Figure 2:**
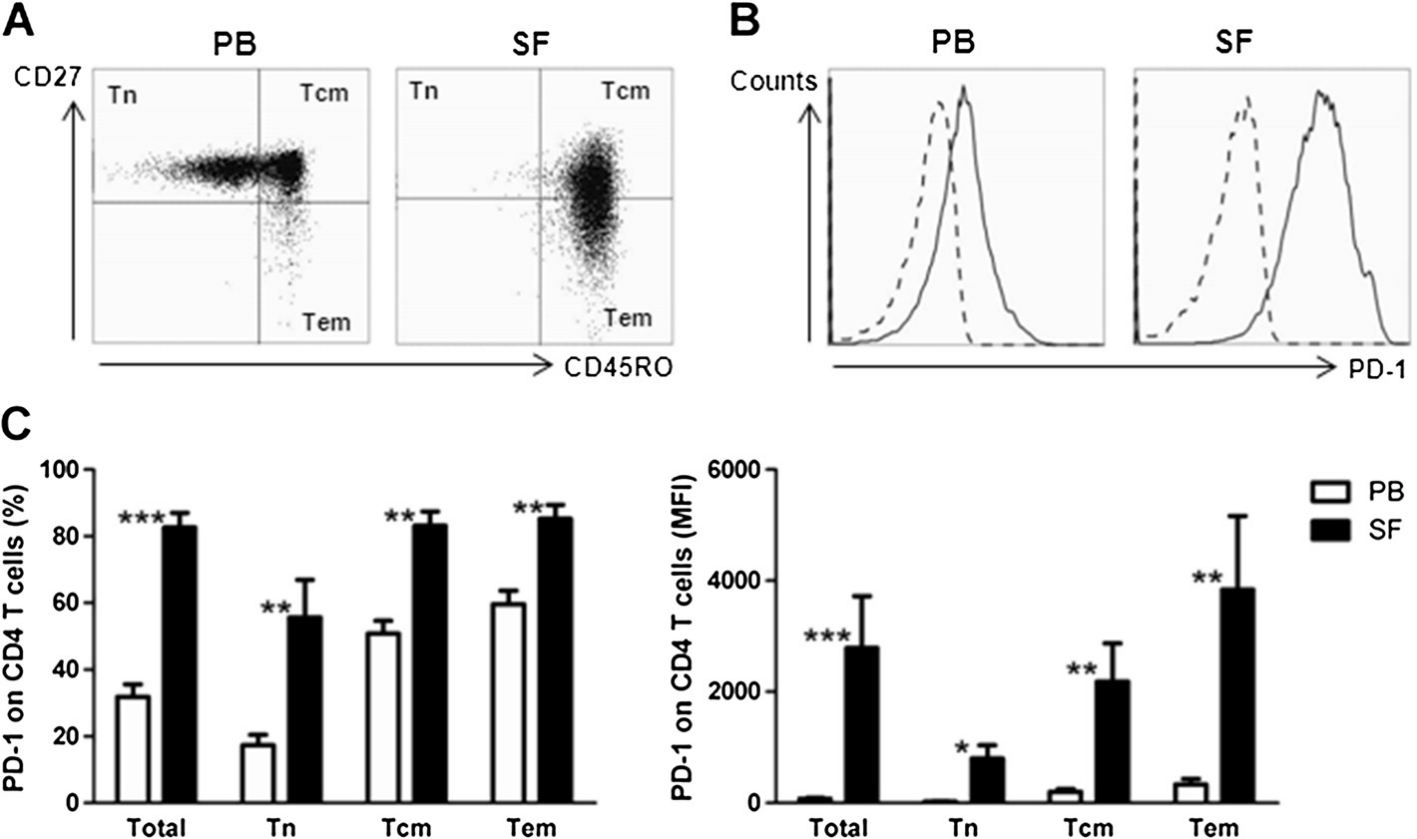
**Memory CD4 T cells, in particular synovial fluid–derived T cell subsets, express increased PD-1 levels.** **(A)** CD4 T cells derived from synovial fluid (SF) of rheumatoid arthritis (RA) patients hardly contain any naïve T (Tn) cells and **(B)** express increased PD-1 levels. Representative histogram of PD-1 expression on PB and SF CD4 T cells of one donor is shown. Dashed line represents autofluorescence. **(C)** PD-1 expression is increased on central memory (Tcm) and effector memory (Tem) T cells in comparison with naïve T cells (Tn cells) from peripheral blood (PB; *n* = 9, both *P* < 0.01) and SF (*n* = 7, both *P* < 0.05). PD-1 expression was strongly upregulated on all CD4 T cell subsets derived from SF versus PB of RA patients. *P < 0.05, **P < 0.01 and ***P < 0.001 (statistically significant differences). MFI, mean fluorescence intensity; PD-1, Programmed death 1.

CD4 T cell subsets can be divided into naïve, central memory and effector memory T cells (Tn, Tcm and Tem cells, respectively), based on the expression of CD27 and CD45RO. In RA patients, PB-derived T cells consist mainly of naïve and central memory T cells, whereas SF-derived T cells hardly contain any naïve T cells and consist mainly of central memory and effector memory T cells (Figure 2A). Because SF memory T cell hyporesponsiveness upon TSLP-mDC stimulation can be caused by enhanced expression of PD-1 interactions, we measured the expression of this inhibitory receptor on the CD4 T cell subsets derived from PB and SF of RA patients (Figure 2B, representative histograms). Naïve T cells from PB and SF expressed the lowest levels of PD-1, although naïve CD4 T cells from SF had strongly upregulated levels of PD-1 compared with naïve CD4 T cells from PB (Figure 2C). However, considering the different expression levels of CD45RO on these ‘naïve’ SF T cells compared to those from PB, we are reluctant to characterise these cells as ‘naïve’ (Figure 2A). Mainly memory T cell subsets expressed PD-1, and a robustly increased percentage of Tcm and Tem cells from SF expressed PD-1 compared with those from PB (Figure 2C).

### PD-L1 is upregulated by TSLP and highly expressed on synovial fluid CD1c mDCs

**Figure 3:**
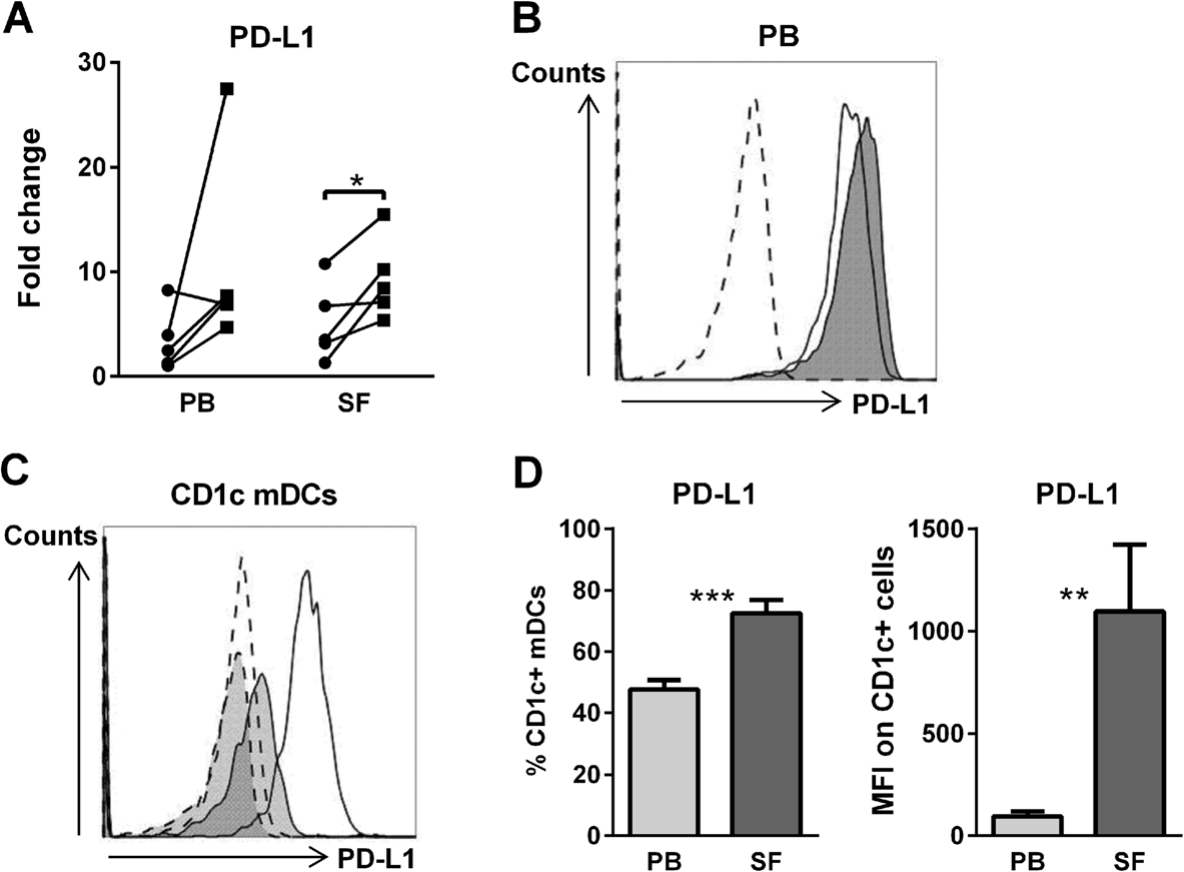
**PD-L1 is upregulated by TSLP and highly expressed on synovial CD1c myeloid dendritic cells.** **(A)** Thymic stromal lymphopoietin (TSLP) stimulation of myeloid dendritic cells (mDCs) significantly upregulates PD-L1 mRNA expression (peripheral blood (PB)–derived and synovial fluid (SF)–derived mDCs of rheumatoid arthritis (RA) patients; circles represent unstimulated mDCs, squares represent TSLP-stimulated mDCs, *n* = 5) and **(B)** PD-L1 protein expression. Representative histrogram of PD-L1 expression on TSLP-stimulated mDCs (filled dark grey area) and unstimulated mDCs (transparent) from PB of one donor is shown. Dashed line represents autofluorescence. **(C, D)** PD-L1 is expressed to a higher extent on CD1c mDCs derived from SF (*n* = 8) compared with PB (*n* = 9) of RA patients. **(C)** Representative histogram of PD-L1 expression on PB mDCs (filled light grey area; solid line) and SF mDCs (transparent area; solid line) of one donor is shown. Dashed line represents autofluorescence for PB mDCs (filled light grey area) and SF mDCs (transparent). **(D)** Mean percentages and MFI of PD-L1 expression of PB and SF mDCs (*n* = 9 and *n* = 8, respectively). *P < 0.05, **P < 0.01, and ***P < 0.001 (statistically significant differences). MFI, Mean fluorescence intensity; PD-L1, Programmed death ligand 1.

Confirming microarray data, TSLP significantly upregulated the mRNA expression of PD-L1 in PB- and SF-derived mDCs (Figure 3A), showing much higher expression levels of PD-L1 mRNA expression compared with PD-L2 (data not shown). The PD-L1 mRNA upregulation upon TSLP stimulation of mDCs was confirmed by an increased PD-L1 surface expression (Figure 3B). To confirm our RNA data, we assessed the *ex vivo* PD-L1 protein expression on CD1c mDCs from PB and SF of RA patients (Figure 3C). The number of mDCs expressing PD-L1 was significantly increased in SF compared with PB, and mDCs derived from SF had a higher intensity of PD-L1 expression compared with mDCs derived from PB (Figure 3D).

### TSLP-mDC driven memory T cell hyporesponsiveness is partially dependent on PD-1/PD-L1 interactions

**Figure 4:**
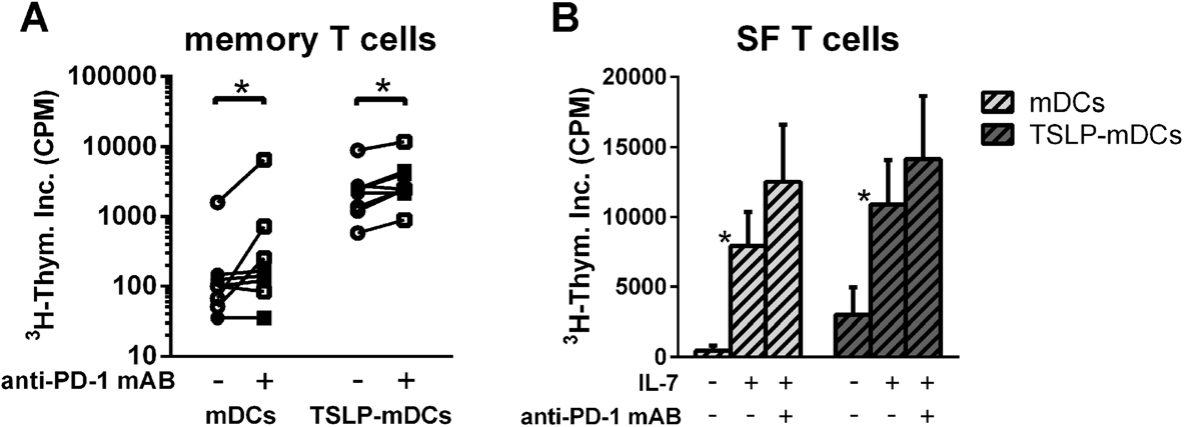
**Hyporesponsiveness of memory CD4 T cells from rheumatoid arthritis patients upon TSLP-primed myeloid dendritic cell stimulation is at least partially reversed by PD-1 blockade. (A)** Blockade of PD-1/PD-L1 interactions by anti-PD-1 antibody (1 μg/ml) in (TSLP-primed) myeloid dendritic cell (mDC) driven memory CD4 T cell activation from peripheral blood (PB) (*n* = 4, filled symbols) and synovial fluid (SF) (*n* = 4, open symbols) partially restores the T cell proliferative capacity. **(B)** Memory CD4 T cell hyporesponsiveness from SF (*n* = 4) is strongly reversed by interleukin-7 (IL-7), a cytokine that largely downregulates PD-1. Additional PD-1 blockade by anti-PD-1 antibody further reversed the downregulated proliferation. Statistical differences between these conditions were assessed using the paired-samples *t*-test. *P < 0.05 (statistically significant difference). PD-1, Programmed death 1; PD-L1, Programmed death ligand 1; TSLP, Thymic stromal lymphopoietin.

Next, we examined whether the hyporesponsiveness of SF memory CD4 T cells was caused by their increased expression of PD-1 in combination with the increased PD-L1 expression on SF-derived mDCs. For this purpose, we cocultured memory CD4 T cells derived from either PB or SF of RA patients with TSLP-activated mDC from PB or SF, respectively, in the presence and absence of PD-1 blocking antibodies. Both memory CD4 T cells from PB and SF showed a similar reduced capacity to respond to TSLP-activated mDCs (data not shown). Blocking PD-1 interactions in cocultures of memory T cells activated by mDCs resulted in a significant increase of the T cell proliferation (Figure 4A). Stimulation of mDCs by TSLP resulted in upregulated memory T cell proliferation, which was also elevated by blocking PD-1 ligation (Figure 4A).

Previously, it has been demonstrated that synovial CD4 T cells are hyperresponsive to IL-7 and that IL-7 has the capacity to downregulate PD-1 expression [18,19]. Therefore, using another way to regulate PD-1 signalling, we assessed the functional consequence of adding IL-7. IL-7 strongly enhanced memory T cell proliferation activated by mDCs or TSLP-mDCs. However, although we observed that IL-7 inhibited PD-1 expression after 20 hours, PD-1 downregulation was apparently not complete, as IL-7-induced T cell proliferation was further enhanced upon prevention of PD-1 signalling by PD-1-blocking antibodies (Figure 4B).

## Discussion

In the present study, we have demonstrated that, in contrast to CD4 T cells from PB, CD4 T cells derived from SF are hyporesponsive upon activation by (TSLP-primed) CD1c mDCs. SF-derived CD4 T cells consist mainly of memory T cells, which express elevated levels of PD-1, whereas SF-derived CD1c mDCs abundantly express its ligand PD-L1, which is upregulated by TSLP. Memory T cell hyporesponsiveness is partially dependent on PD-1/PD-L1 interactions because blockade of these interactions by anti-PD-1 antibodies partially restores the T cell proliferation. Importantly, IL-7, an inhibitor of PD-1 expression, also overcomes hyporesponsiveness and robustly activates synovial memory T cells in the context of activated (TSLP-primed) mDCs, which could be further enhanced by blocking PD-1.

Similarly to TSLP-primed mDCs from PB, SF-derived mDCs have a strongly enhanced capacity to activate T cells derived from PB that is even upregulated upon TSLP stimulation [13]. Associated with this enhanced T cell stimulatory capacity, enhanced expression of antigen-presenting molecules, including CD1c and HLA class II, and costimulatory molecules, including CD80, CD86 and CD40, have been demonstrated [11,13]. In support of this finding, activation of PB-derived T cells by (TSLP-primed) mDCs is strongly dependent on costimulation and antigen presentation as blockade of HLA-class II molecules or CD80/86 costimulation blocked T cell activation [13]. However, despite the abundance of antigen-presenting and costimulatory molecules, the activity of T cells derived from SF of RA patients activated by autologous (TSLP-primed) mDCs is strongly hampered. Although several mechanisms might be involved in the reduced capacity of SF-derived T cells in response to activated mDCs, the role of PD-1/PD-L1 interactions, which negatively regulate T cell activation [15], was investigated for obvious reasons, such as overexpression of PD-L1 by activated mDCs from SF and high PD-L1 mRNA levels induced by TSLP. Blockade of PD-1/PD-L1 interactions has been shown to result in increased T cell activity [23] and PD-1 deficiency, and PD-L1 deficiency is shown to play a critical role in the development of autoimmune disorders in mice [24,25].

In line with our results showing increased PD-1 expression on SF-derived memory T cells compared with PB-derived T cells, PD-1 expression was previously described to be increased on SF-derived CD4 T cells [17]. These SF-derived T cells were previously suggested to be resistant to PD-1 mediated suppression, as PD-L1 expression on SF-derived CD14+ monocytes and macrophages was not sufficiently high to effectively downregulate T cell activation [17]. In contrast to this latter suggestion, we observed hyporesponsiveness of SF T cells upon activation by *in vivo* activated synovial mDCs and *in vitro* activated TSLP-mDCs, which, in contrast, expressed high levels of PD-L1. PD-1-blocking antibodies, blocking interactions with PD-L1, resulted in an increase in the T cell proliferation, indicating that SF-derived T cell hyporesponsiveness is at least partially dependent on PD-1/PD-L interactions. However, based on the strong expression of both PD-1 on T cells and PD-L1 on activated mDCs, more robust reversal of T cell hyporesponsiveness was expected. The lack of response might be due to the fact that PD-1 is also expressed on regulatory T cells, preventing immunosuppression by these cells [26]. Because previous studies, including those of our group, have demonstrated an increased presence of regulatory T cells in the SF [27], blockade of PD-1, next to increased activation of effector cells, might enhance the function of regulatory T cells, resulting in a somewhat more limited reversal of T cell inhibition. An alternative explanation is that enhanced antigen presentation due to robust upregulation of HLA class II by TSLP may limit the effect of PD-1 blockade, which is consistent with previous data demonstrating that the inhibitory effect of the PD-1/PD-L pathway on CD4 T cells was greatest at lower antigen concentrations [16,25]. Additionally, blockade of PD-L1 has been shown to more potently restore T cell proliferation compared with PD-1 blockade in antigen-driven T cell responses [28]. Although not tested in the present study, this might result in a stronger restoration of the SF-derived T cell hyporesponsiveness.

Hyporesponsiveness of SF-derived CD4 T cells upon mDC or TSLP-mDC stimulation was robustly reversed by IL-7, which is in line with previous data demonstrating vigorous activation and even hyperresponsiveness of synovial CD4 T cells in the context of monocytic cells [19]. Recently, IL-7 was shown to prevent shutdown of T cell activation by downregulation of PD-1 expression [18]. We also found PD-1 downregulation upon IL-7 stimulation of CD4 T cells in the present study (data not shown). Apart from the capacity of IL-7 to downregulate PD-1 expression [18], IL-7 is also shown to overcome PD-1-mediated inhibition of T cells by STAT5 activation that is regulated by IL-2 and family members such as IL-7 and IL-15 [29,30]. IL-7 and IL-15 are both abundantly present in joints of RA patients [19,31], which suggests that the inflammatory environment in RA joints could play a critical role in overcoming PD-1-mediated inhibition of T cells. Although reversing PD-1-mediated inhibition is likely a crucial mechanism whereby IL-7 overcomes hyporesponsiveness, other mechanisms induced by IL-7 could be involved as well, such as upregulation of (co)stimulatory molecules that were previously found to be induced by IL-7, both on T cells (for example, lymphocyte function–associated antigen and CD69) and on myeloid cells (CD40, CD80, CD86) [19].

## Conclusions

The present study demonstrates that PD-1/PD-L1 interactions between SF-derived T cells and *in vivo* activated SF mDCs or *in vitro* TSLP-primed mDCs contribute to T cell hyporesponsiveness. The potential of IL-7 to robustly counteract this hyporesponsiveness suggests that such cytokines in joints of RA patients might strongly contribute to the activation of memory T cells, in addition to the potential of SF-derived mDCs to activate autologous peripheral (naïve) CD4 T cells attracted to the joint.

## Abbreviations

BDCA: Blood-derived dendritic cell antigen
DC: Dendritic cell
FACS: Fluorescence-activated cell sorting
IL: Interleukin
mDC: Myeloid dendritic cell
MNC: Mononuclear cell
PB: Peripheral blood
PD-1: Programmed death 1
PD-L: Programmed death ligand
RA: Rheumatoid arthritis
RT-qPCR: Quantitative reverse transcription polymerase chain reaction
SF: Synovial fluid
Tcm: Central memory T cell
Tem: Effector memory T cell
Tn: Naïve T cell
TSLP: Thymic stromal lymphopoietin
